# Supplementary material for: Temporal network analysis of gut microbiota unveils aging trajectories associated with colon cancer
Source: mSystems. 2025 Apr 29;10(5):e01188-24. doi: 10.1128/msystems.01188-24 (PMC12090783; doi:10.1128/msystems.01188-24)
Supplement: Supplemental figures — Figures S1 to S4. [file msystems.01188-24-s0001.pdf]

Supplemental figure

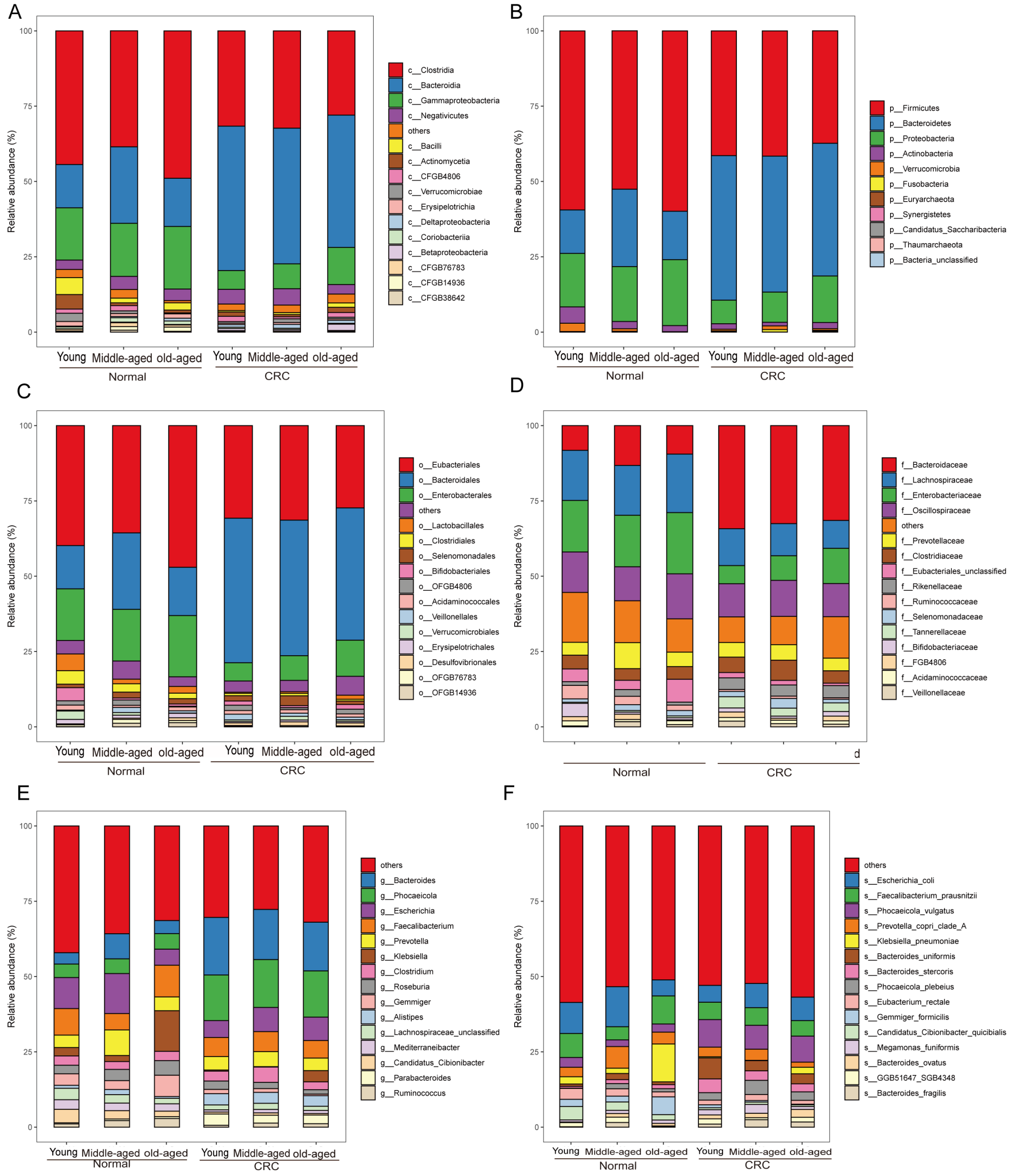

Supplemental figure1. Species composition at different levels. (A) Species composition at the phylum level for the top 15 species in each group. (B) Species composition at the class level of the top 15 Bacteroidetes for each group. (C) Species composition at the order level of the top 15 Bacterial Orders for each group. (D) Species composition at the family level of the top 15 Bacterial families for each group. (E) Species composition at genus level for the top 15 species of each group. (F) Species composition at the species level of the top

15 bacterial species in each group.

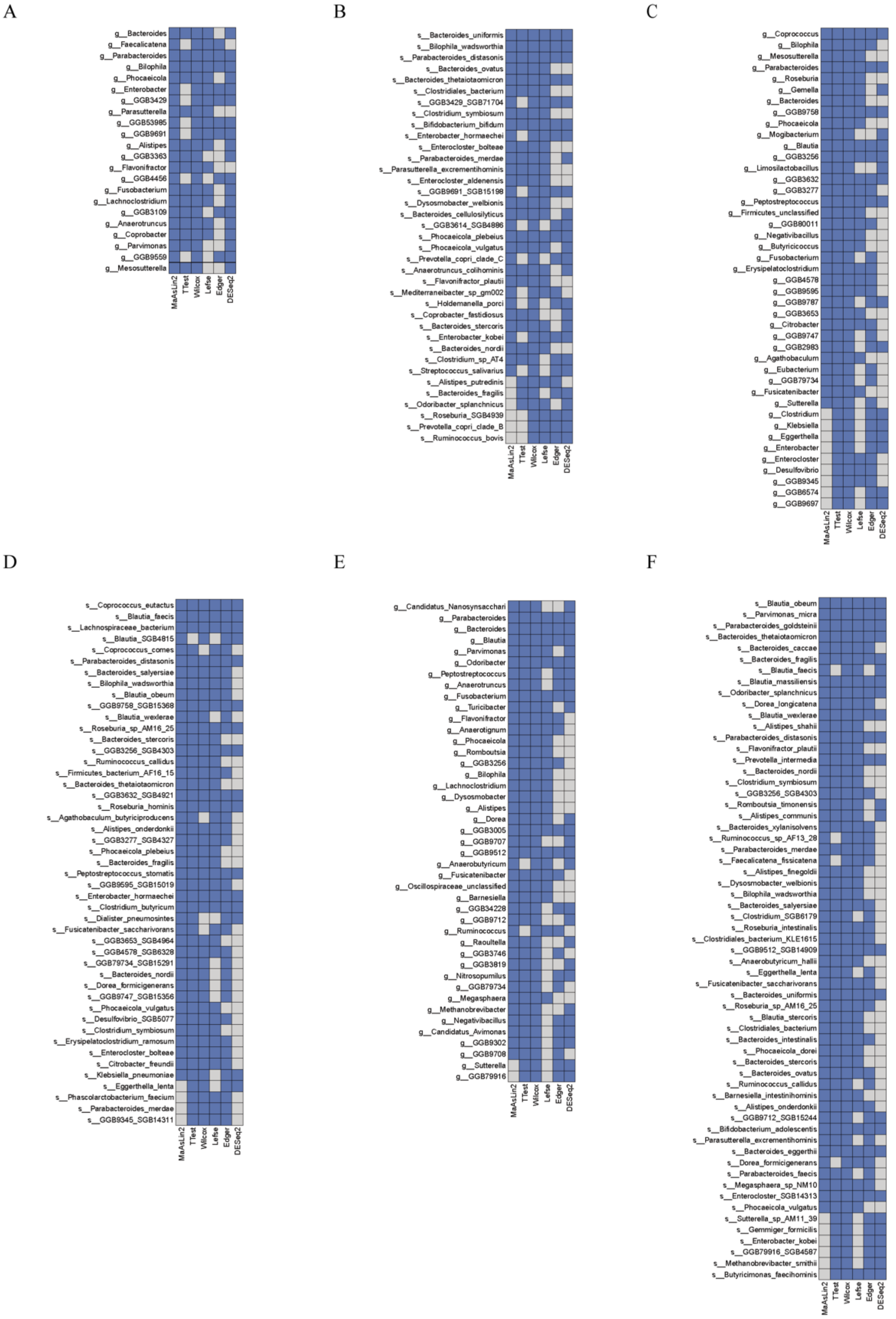

Supplemental figure2. Heatmap of 6 different methods of analyzing differences at different levels. (A) Heatmap of 6 different methods of analyzing differences in genus levels between the young CRC group and the young healthy

group. (B) Heatmap of 6 different methods of analysis of variance at genus level for the middle- aged CRC group versus the middle-aged health group. (C) Heat map of 6 different methods of analysis of variance at the genus level for the older CRC group versus the older health group. (D) Heat map of 6 different methods of analysis of variance at genus level for the Young CRC Group versus the Young Healthy Group. (E) Heat map of 6 different methods of analyzing differences between the middle- aged CRC group and the middle-aged health group at species level. (F) Heat map of 6 different methods of analysis of variance for the elderly CRC group versus the elderly health group at the species level.

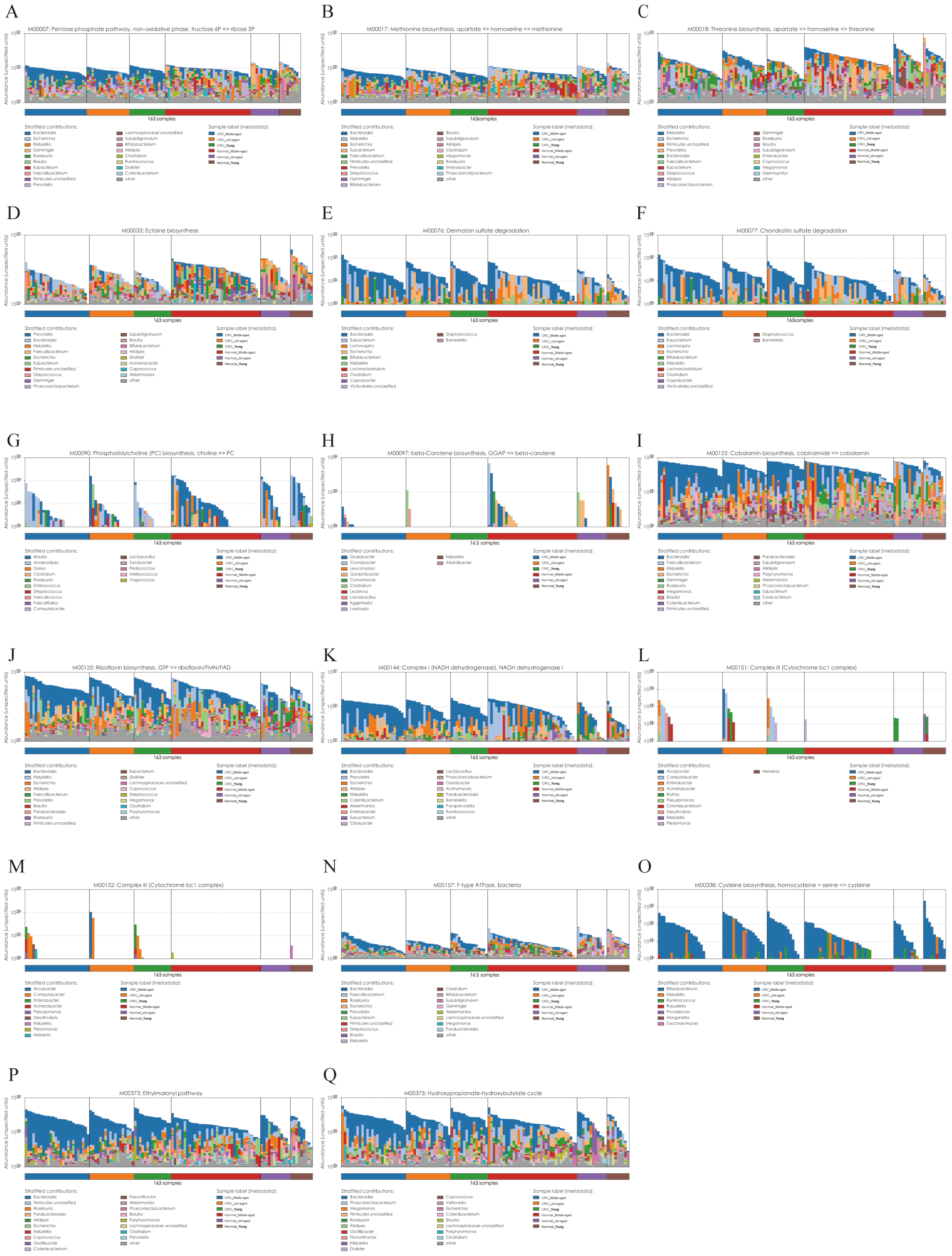

Supplemental figure3. Stratified histogram of (A-Q) species level difference moudel.

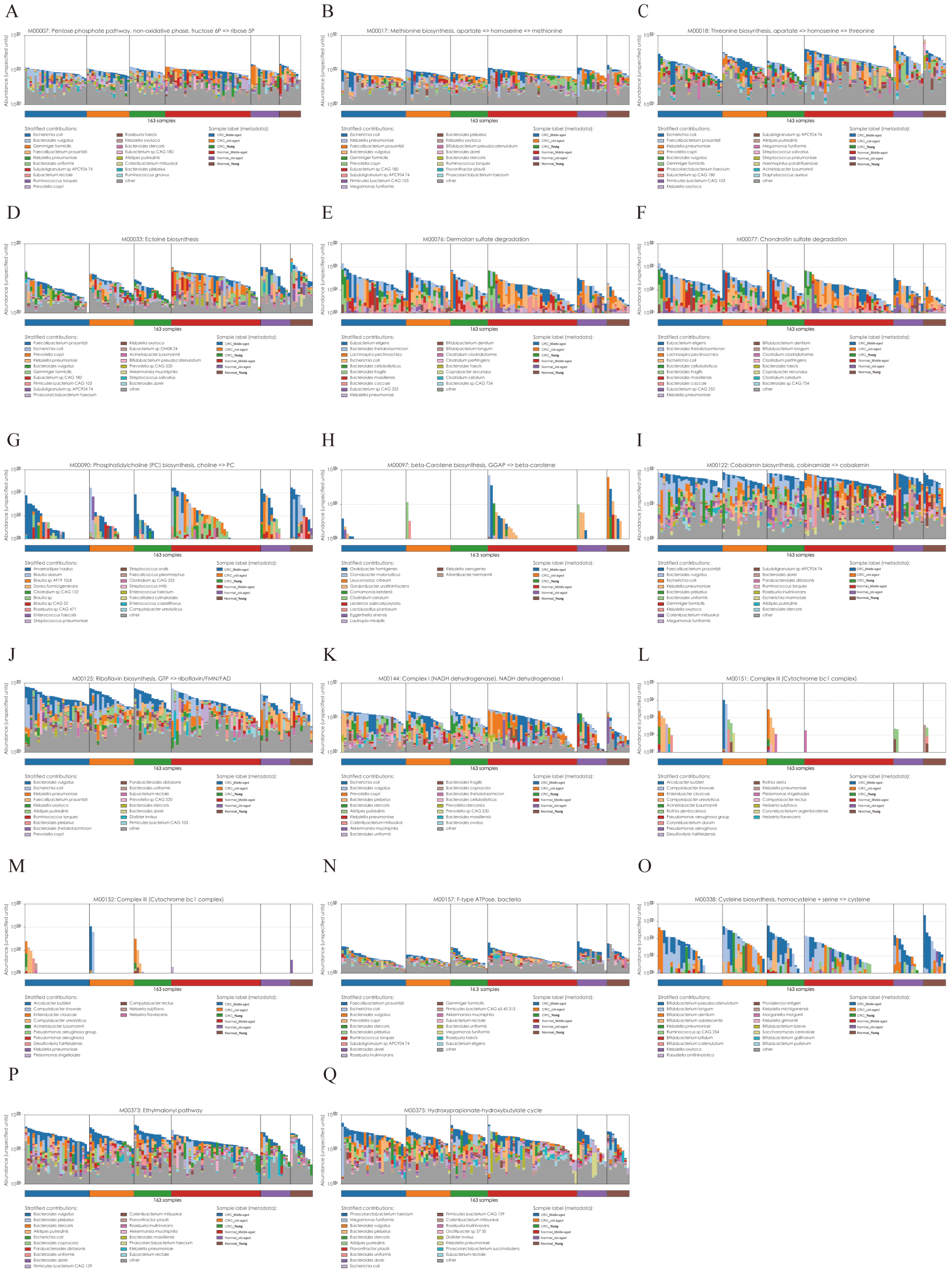

Supplemental figure4. Stratified histogram of (A-Q) genus level differences moudel
